# Myofibroblast Gene Expression Profile after Tooth Extraction in the Rabbit

**DOI:** 10.3390/ma12223697

**Published:** 2019-11-09

**Authors:** Simone Marconcini, Maria Denaro, Saverio Cosola, Mario Gabriele, Paolo Toti, Eitan Mijiritsky, Agnese Proietti, Fulvio Basolo, Enrica Giammarinaro, Ugo Covani

**Affiliations:** 1 Tuscan Dental Institute, Versilia General Hospital, 55041 Lido di Camaiore, Italy; s.cosola@hotmail.it (S.C.); capello.totipaolo@tiscali.it (P.T.); e.giammarinaro@gmail.com (E.G.); 2 Department of Surgical, Medical, Molecular Pathology and Critical Area, University of Pisa, 56124 Pisa, Italy; mariadenaro.md@libero.it (M.D.); mario.gabriele@unipi.it (M.G.); f.basolo@med.unipi.it (F.B.); covani@covani.it (U.C.); 3 Department of Otolaryngology Head and Neck Surgery and Maxillofacial Surgery, Tel-Aviv Sourasky Medical Center, Sackler School of Medicine, Tel Aviv 61503, Israel; mijiritsky@bezeqint.net; 4 Section of Surgical Pathology, University Hospital of Pisa, 56124 Pisa, Italy; agneseproietti@gmail.com

**Keywords:** wound healing, gene expression, fibroblasts, alveolar bone

## Abstract

After tooth extraction, the alveolar bone tends to shrink in volume, especially on the vestibular side. The role of myofibroblasts in bone remodeling has not been sufficiently investigated. The aim of the present study was to explore the gene expression related to myofibroblasts presence and activity during a 90-day healing period after tooth extraction. The study included 36 rabbits, and a single tooth extraction was performed on each rabbit. The extractive sockets were randomly distributed to natural healing or to scarification of the wound. The sacrifices were staggered in such a manner that animals contributed with sockets representing 2, 7, 15, 30, 60, and 90 days of healing. Nanostring technology was used to evaluate the expression of a wide panel consisting in 148 genes related to the activation, induction, and suppression of myofibroblasts, socket microenvironment, and autophagy. We found that the expression profile of this custom panel was time-related. The post-extractive socket was subjected to significant gene expression changes after 15 days: the genes involved in the induction of myofibroblasts were up-regulated in the first 15-day period and down-regulated during the rest of the follow-up. The study suggested that myofibroblasts play a major role in the immediate 15-day period following tooth extraction.

## 1. Introduction

Tooth extraction entails large remodeling processes that end in significant bone resorption of 50% in volume within a three- to six-month period, this resorption occurring predominantly at the buccal aspect of the ridge [1,2,3]. This consistent phenomenon poses a risk for future rehabilitation, as local unfavorable hard and soft tissues anatomy might prevent dental implant placement or, at least, impair the overall esthetic outcome [4]. Covani and colleagues showed that after single tooth extraction, the alveolar crest tends to move two-thirds lingually/palatally from the original buccal edge, the shift occurring predominantly at the geometrical midpoint of the edentulous site (64.8% ± 10.5% of the distance from the vestibular margin to the crest) [5]. 

Bone remodeling takes place on both buccal and lingual walls, but given the fact that the lingual bone plate is thicker, the three-dimensional remodeling results in greater loss at the thin buccal plate with respect to the wide lingual wall [6].

Although the histological sequence of alveolar socket healing has been described in depth [7], the influence of local geometry upon connective tissue healing is largely unknown. The early post-extractive alveolus might be described as a wound that runs through three sequential phases: inflammatory, proliferative, and modeling/remodeling. During the inflammatory phase, the combination of inflammatory cells, vascular sprouts, and immature fibroblasts forms the granulation tissue. As the site becomes sterilized, the granulation tissue is gradually replaced with a provisional connective tissue matrix rich in collagen fibers and cells, and the proliferative phase begins. 

The evolution of the granulation tissue between the socket walls is carried out by fibroblasts (FB), ubiquitous cells that are normally mechanically stress-shielded by the collagen architecture of intact connective tissues [8,9]. Tissue injury, i.e., the interruption of tissue contiguity, directly exposes fibroblast receptors to mechanical stress, initiating a repair cascade aiming to restore the mechanical tissue integrity. Local fibroblasts become reparative myofibroblasts (MFB) with a contractile phenotype: in order to fill in the damaged tissue, fibroblasts acquire a migratory phenotype by means of de novo production of contractile bundles that develop small traction forces [10]. This initial activated FB might be named a “proto-myofibroblast”, and it is promoted by changes in the properties of the extracellular matrix (ECM) and by local release of inflammatory cytokines such as tumor necrosis factor alpha (TNF-**α**) [11]. Their own activity increases the stiffness of the ECM, which in turn promotes a further cellular differentiation to proper MFB expressing alpha smooth muscle actin (α-SMA) in a positive feedback loop. Myofibroblasts are extracellular-matrix-secreting cells and are largely responsible for the contractility of scar tissue as it matures over time. Myofibroblasts, like smooth muscle cells and fibroblasts, develop contractile force upon phosphorylation of myosin light chains, which allows the myosin head to interact with actin filaments [12]. Mechanical challenges represent the main factor determining connective cells features: the stiffness of the ECM modulates cell proliferation, differentiation, migration, and gene expression [13].

The healing response is regulated by signaling molecules (i.e., growth factors and cytokines): they initiate cell migration, differentiation, and proliferation as they interact with each other in highly ordered temporal and spatial sequences [14]. The molecular stigmata of myofibroblast activity have been typified in several pathological conditions, such as under fibrotic conditions of the lungs [15], heart [16], and gingival tissue fibromatosis [17]. Soft-tissue-specific myofibroblasts are difficult to distinguish from endothelial myofibroblasts in experimental models since both of them express the same α-SMA.

Fibroblasts populating the granulation tissue of a wound that was mechanically stressed by splinting with a plastic frame formed more stress fibers, and therefore, proto-myofibroblasts would appear earlier than in natural wound healing [18]. The role of mechanical stress in stimulating myofibroblast activity has also been shown in experiments where dermal wounds in mice were mechanically stressed by stretching or splinting the wound, where increased myofibroblast activity was observed. 

Unlike most other adult tissues, but similar to embryonic ones, oral gingiva and oral mucosa scar only a little upon injury. 

Our hypothesis is that myofibroblasts might have a crucial role during the healing of tooth extraction sockets, possibly determining the overall remodeling pattern of the alveolar bone. Using a sensitive and high-throughput method, the aim of the present molecular study was to explore the gene expression profile related to myofibroblast activation and local microenvironment changes during a 90-day period after tooth extraction and whether this activation was dependent on the type of surgical procedure or on a time-related factor. The present study is part of a larger upcoming report including bone block section evaluation via microcomputed tomography.

## 2. Materials and Methods

### 2.1. Study Design

The study design was developed in accordance with internationally accepted ARRIVE guidelines that were intended to improve the reporting of research using animals. The protocol included 36 rabbit models. The sample size was chosen according to previous published literature and following the ethical principle of minimum sacrifice but sufficient power. A randomization schedule was obtained using statistical software. One lower incisor in each rabbit was extracted, and then the socket was randomized to two different managements. In the first group, the interrupted soft tissues were adjoined with tension-free stitches (Group 0). In the second group, the extraction socket was filled with a collagen sponge and the surrounding soft tissues were partly disrupted with the surgical blade in order to create the conditions for secondary intention healing (Group 1). In the second step of the investigation, the animal models were divided into six groups according to the time between the tooth extraction and the post-extractive socket sampling. Each group included six samples and was indicated by T1 to T6. The six time-related groups corresponded respectively to 2, 7, 15, 30, 60, and 90 days after tooth extraction. 

### 2.2. Animal Model and Management

Study approval was obtained from the Ethical Commission for Animal Welfare, Pisa, Italy (IRB 0035123/2017). Thirty-six white adult male New Zealand rabbits with an average body weight of 2 kg were purchased and housed in an enclosure at the Veterinary Department of the University of Pisa. The person in charge of the welfare of the animals took care of aeration and food and water administration, as well as the animals’ behavioral and health conditions throughout the study period. All animals were pre-medicated with an intramuscular injection of 0.2 mL meloxicam (Metacam, Boehringer Ingelheim, Ingelheim am Rhein, Mainz-Bingen district, Rhineland-Palatinate state, Germany, 0.5 mg/kg). On the day of surgery, all animals were anesthetized according to the following procedure: 0.8 mL of intramuscular alphaxolone (Alfaxan; Jurox UK, Worcestershire, United Kingdom, 10 mg/mL). An additional local anesthesia (Xylocain Dental adrenalin, Astrazencea, Milano, Italy, 20 mg/mL + 12.5 mg/mL) was given to reduce the dosage of the systemic anesthetic as well as to reduce the bleeding during surgery and to alleviate pain after surgery. Postsurgical treatment with systemic antibiotics (Baytril, Bayer S.p.A., Milano, Italy, 25 mg/mL) was given for five days to avoid infections. Within the first days after surgery, all animals were monitored routinely and further analgesia was given if necessary. The whole study was accompanied and monitored by a veterinarian, and surgeons with extensive experience performed all surgical procedures. 

### 2.3. Surgical Phase 

The two surgical procedures were performed under aseptic conditions in an animal operating theater under general anesthesia. The lower right incisor was carefully extracted, and then the site was assigned to primary intention healing with tension-free suture (Group 0) or to second intention healing with tissue scarification and filling of the socket with a sterile collagen sponge (Gingistat, Vebas) (Group 1). Postoperatively, the wounds were inspected daily for eventual clinical signs of complications. Checkups were performed on a regular basis throughout the experiment. 

### 2.4. Terminal Procedure 

Thirty-six animals were sacrificed in groups of six at 2, 7, 15, 30, 60, and 90 days after tooth extraction. The termination was conducted by inducing respiratory arrest with an intravenous injection of a 20% solution of pentobarbital. 

### 2.5. Histological Preparation

**Figure 1:**
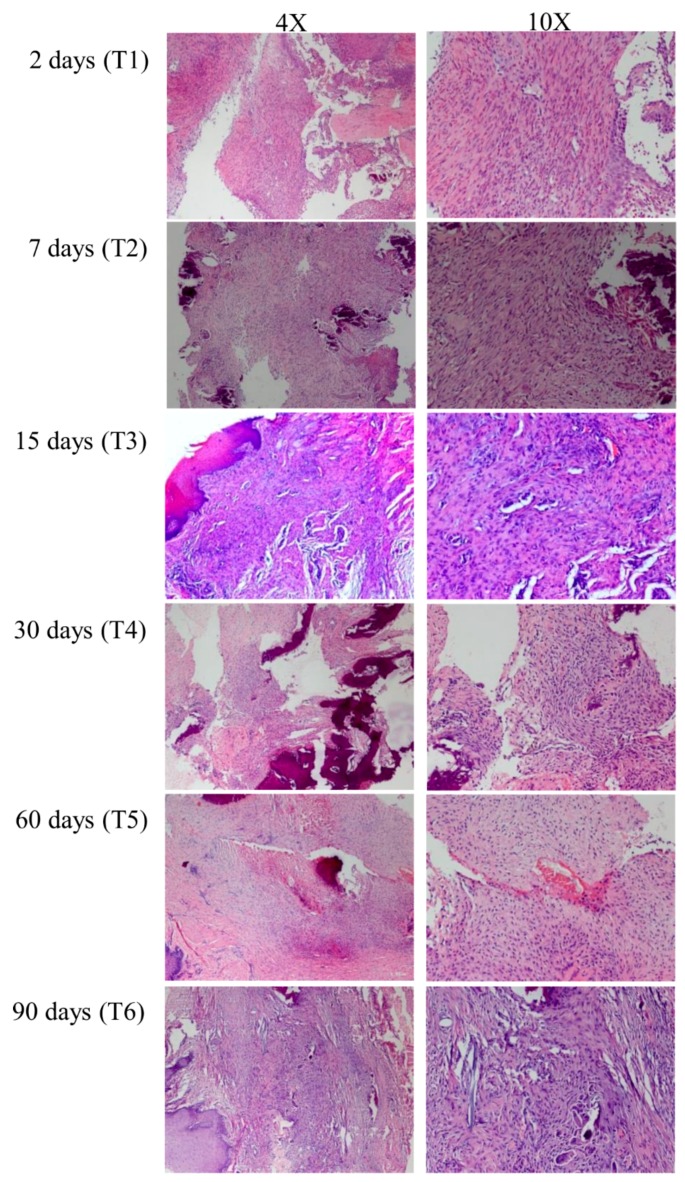
Hematoxylin and eosin stained histological sections of representative sockets at 2 (T1), 7 (T2), 15 (T3), 30 (T4), 60 (T5), and 90 (T6) days post-extraction. For each group, 4 × and 10 × magnifications are reported.

Block resections of the extraction sites were performed using an oscillating autopsy saw to keep the soft tissue intact. The operator carefully detached the granulation soft tissue from the underlying alveolar bone in a full-thickness fashion. All tissues were formalin fixed and paraffin embedded. Two-micrometer-thick sections were stained with hematoxylin and eosin and prepared for microscopic examination (Olympus BX51, Olympus Italia, Segrate, MI). In each sample, tissue areas with extensive young fibrosis rich in myofibroblasts were selected for the following RNA purification (Figure 1). Bone samples were stored for future micro-CT analysis, the results of which are not an object of the present study.

### 2.6. RNA Purification

Tissue sections with a thickness of 5 μm underwent standard deparaffinization. Myofibroblast-rich areas were localized, and the total RNA was purified using a Qiagen RNeasy FFPE kit (Qiagen, Hilden, Germany) according to the manufacturer’s instructions. The total RNA concentration was assessed using an Xpose spectrophotometer (Trinean, Gentbrugge, Belgium).

### 2.7. nCounter Nanostring Technology

nCounter NanoString technology was used for a simultaneous digital detection of the target mRNA transcripts. This methodology is based on direct molecular barcoding of target molecules through the use of specific probe pairs without the use of reverse transcription or amplification. A total of 150 ng of RNA was added to the capture and reporter probes in each hybridization reaction. Hybridization was performed for 18 h at 65 °C in a SensoQuest thermal cycler (SensoQuest, Gottingen, Germany). The clean-up of the samples, immobilization on the cartridge, and digital count were performed as described by the manufacturer’s instructions on the prep station and on the Nanostring systems digital counter (NanoString Technologies, Seattle, WA, USA).

### 2.8. nCounter Custom Panel 

The nCounter custom gene expression panel was designed by the authors of the present study after a literature search for myofibroblast-related genes, autophagy-related genes, and genes coding for oral mucosa and socket microenvironment components [19,20,21]. The custom panel was synthesized using Nanostring technology (Nanostring Technologies, Seattle, WA, USA) and included a total of 163 probe pairs directed against 148 target genes and 15 housekeeping genes. In more detail, the panel consisted of genes related to the activation, induction, and suppression of myofibroblasts; autophagy; growth factors and cytokines; extracellular matrix (ECM) components; surface proteins; and oral mucosa (Table 1).

### 2.9. Gene Expression Analysis

A total of 150 ng of purified RNA was used as the input material for the gene expression analysis using Nanostring technology. According to NanoString recommendations, a 260/280 ratio of 1.9 or greater and a 260/230 ratio of 1.8 or greater were necessary to obtain optimal results.

Technical and biological normalization of the raw counts of each gene was performed using nSolver Software version 2.5 (NanoString Technologies, Seattle, WA, USA). 

For the technical normalization, a positive control factor was calculated for each sample. A positive control factor value outside the range of 0.3–3 indicated technical problems and the subsequent exclusion of the sample from further analysis. At the same time, a biological normalization factor was determined for each sample. The sample was excluded if this value was outside the range of 0.1–10.0. All the normalization steps were performed using Nanostring nCounter software analysis (NanoString Technologies, Seattle, WA, USA).

### 2.10. Statistical Analysis

Mann–Whitney *U*-test followed by Benjamini–Hochberg correction (false discovery rate, FDR) was used to identify differentially expressed genes between Group 0 and Group 1 (adjusted *p*-values of <0.05). 

Kruskal–Wallis test, Dunn’s test, and Bonferroni’s correction were used to identify the differentially expressed genes among the different injury recovery times.

Hierarchical clustering analysis was performed with nSolver Analysis software 2.5 using Pearson’s correlation.

## 3. Results

Overall, RNA purification was conducted on post-extractive socket samples from 36 rabbit models—18 samples belonging to Group 0 and 18 to Group 1. Out of the 36 samples, 33 were appropriate for the gene expression analysis, whereas 3 out of the 36 post-extractive socket samples (2 samples codified as T3-0 and 1 sample as T5-1) were excluded for their inadequate RNA concentrations.

### 3.1. Gene Expression Profile of Post-Extractive Sockets 

To evaluate the gene expression profile of the post-extractive sockets, unsupervised hierarchical clustering analysis was performed including all the 148 target genes of the custom panel. 

This analysis allowed us to identify two main expression profiles constituted by Cluster 1 and Cluster 2. In more detail, Cluster 1 included 18 samples; 8 were sockets from Group 1 and 10 were from Group 0. However, the majority of these samples belonged to the T4, T5, and T6 groups (6 were of the T4 group, 5 of the T5 group, and 6 of the T6 group).

**Figure 2:**
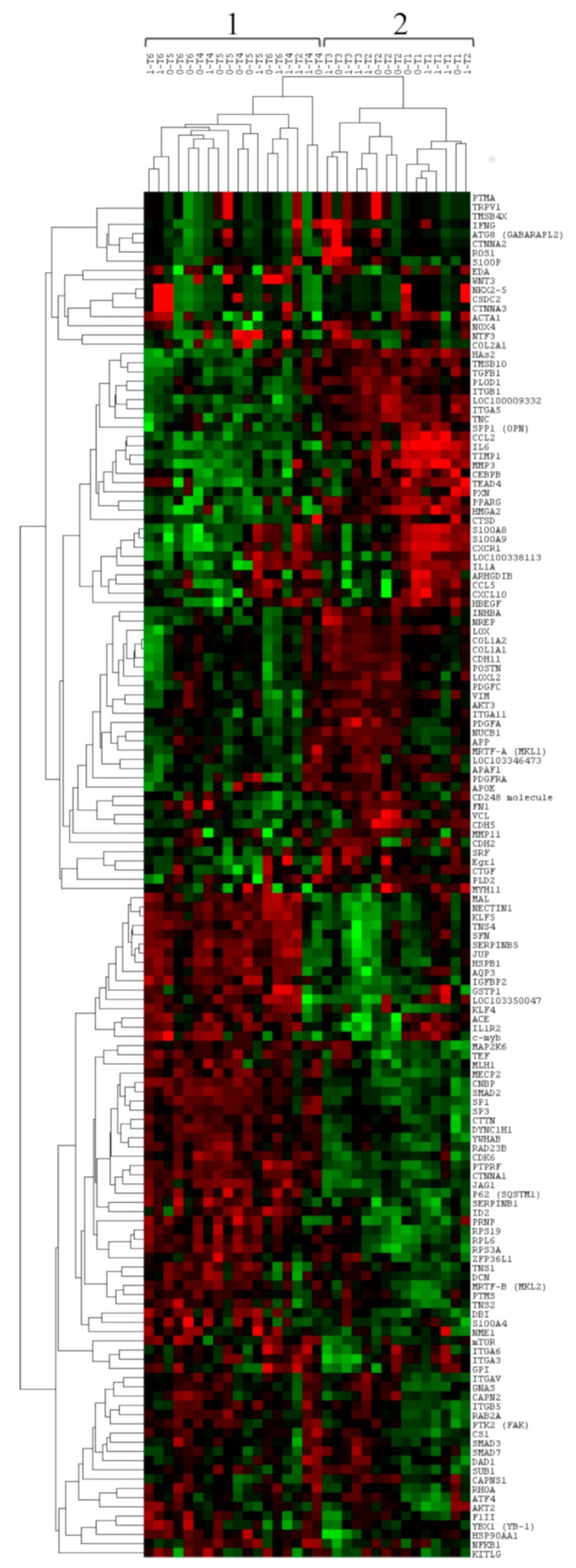
Unsupervised hierarchical clustering analysis of post-extractive sockets using the 148 genes of the custom panel. The two main clusters are indicated with numbers 1 and 2.

Cluster 2 included 15 samples; 8 were sockets from Group 1 and 7 were from Group 0. All the samples belonged to the T1, T2, and T3 groups. Figure 2 shows a dendrogram of the clustering analysis. 

The columns represent the samples and the lines represent the genes. Red color indicates high gene expression levels; green color indicates low gene expression levels.

### 3.2. Comparison of Gene Expression between Group 0 and Group 1

Mann–Whitney *U*-test, followed by Benjamini–Hochberg correction (adjusted *p*-value of <0.05), was used to identify differentially expressed genes between Group 0 and Group 1. According to these analyses, none of the 148 genes were statistically significant between the two analyzed groups.

### 3.3. Comparison of Gene Expression among Time-Related Groups

Kruskal–Wallis test, Dunn’s test, and Bonferroni’s correction were used for multiple comparisons among the time-related groups between the tooth extraction and the post-extractive socket sampling. 

The differentially expressed genes among the analyzed groups are reported in Table 2, Table 3, Table 4, Table 5, Table 6, Table 7, Table 8 and Table 9. No genes were significantly differentially expressed in the comparisons of T2 vs. T3, T4 vs. T5, T4 vs. T6, and T5 vs. T6; for this reason, they are not reported in the tables. 

In more detail, the statistically significant genes involved in the activation and induction of myofibroblasts (Table 2 and Table 3) were up-regulated in the first period (2–15 days) after tooth extraction when compared to the rest of the follow-up.

On the contrary, the majority of genes coding for surface proteins (Table 4), constitutive proteins of oral mucosa (Table 5), ECM components (Table 6), growth factors and cytokines (Table 7), and autophagy-related genes (Table 8) exhibited down-regulation in the early healing phase. Particularly, autophagy-related genes were already significantly down-regulated in the first two days (T1 group) after tooth extraction. 

Greater variability was evident for the genes coding for proteins deregulated at 48 h post-surgery (Table 9). While *APOE, ID2*, and *MAL* were down-regulated, the *CXCR1, MMP3*, and *TIMP1* genes were up-regulated in the early phase compared to late-phase healing.

**Figure 3:**
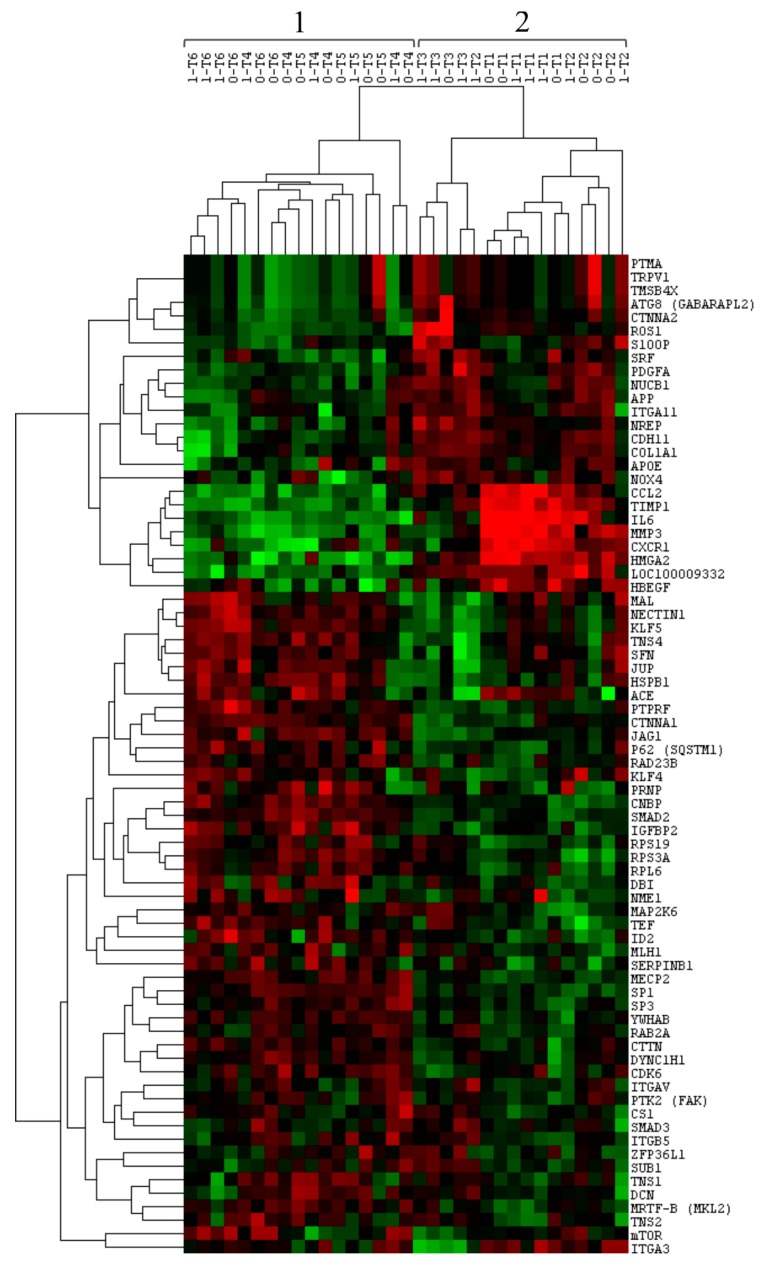
Hierarchical clustering analysis of post-extractive socket samples using the 74 differentially expressed genes from the custom panel. The two main clusters are indicated with the numbers 1 and 2. The columns represent the samples, and the lines represent the genes. Red color indicates high gene expression levels; green color indicates low gene expression levels.

In order to compare the gene expression profiles of the post-extractive socket samples, we performed clustering analysis using the 74 genes differentially expressed among the time-related groups (Figure 3). 

This analysis revealed two main clusters. Cluster 1 included samples belonging to the T4, T5, and T6 groups; Cluster 2 constituted samples belonging to the T1, T2, and T3 groups. In other words, the samples were split into two clusters according to a specific time period: post-extractive sockets collected between 2 and 15 days since the tooth extraction (T1, T2, and T3) were included in Cluster 2; post-extractive sockets that were collected between 30 and 90 days since the tooth extraction (T4, T5, and T6) were included in Cluster 1. 

These results showed that the expression of myofibroblasts and the expression of genes coding for factors of the post-extractive socket microenvironment are modulated during injury recovery and are time-related. In particular, the genes involved in the activation and induction of myofibroblasts were up-regulated in the first 15-day period and down-regulated during the rest of the follow-up. 

## 4. Discussion

The post-extractive dental alveolus is an outlier among second intention wounds—a non-homogeneous one. In fact, early bone healing events in human extraction sockets are characterized by the participation of different cell types, including pericytes, adipocytes, periodontal ligament fibroblasts, marrow stem cells, and periosteal cells [22]. The migration of fibroblasts into and through the extracellular matrix during the initial phase of post-extractive socket healing appears to be a fundamental component of wound contraction. In the context of fibroblast-migration-driven wound contraction, the location and the force generation mechanisms are of central concern^19^. A salient paradigm for connective tissue remodeling is “local geometry regulates cells function” [23]: during the healing process, the flux of fibroblasts reorganizes collagen fibers, and secondly, collagen will align with tension lines in response to tissue displacements. 

To the best of our knowledge, this is the first study performing multiplex expression analysis of myofibroblast-related genes and of genes coding for factors of the socket microenvironment after tooth extraction. The gene expression profile obtained using a custom panel constituted 148 target genes differentiated substantially between the first 15 days and the rest of the follow-up, suggesting that myofibroblasts and the socket microenvironment have different functions during the early and the late healing phases. When we performed clustering analysis using the 74 differentially expressed genes, two homogeneous groups were observed: the first one included samples collected in the first 15-day period from the tooth extraction, and the second one included socket samples collected during the rest of the follow-up. Those findings might give us a clue of the molecular events occurring at the granulation tissue in the early post-extractive socket.

In detail, the present tooth extraction model demonstrated that myofibroblast-related gene expression is markedly modulated during socket injury recovery and that it is time-related. Genes related to the induction and activation of myofibroblasts were up-regulated in the first 15-day period and down-regulated during the rest of the follow-up irrespective of the surgical procedure performed; thus, myofibroblasts play a major role in the early stages of socket healing.

This finding confirmed the classic evidence on wound healing which suggests that myofibroblasts disappear when overlying epithelial closure is achieved, this event usually occurring after 15 days in the oral mucosa. In the resolution phase of healing, the cell number is dramatically reduced by apoptosis of both vascular cells and myofibroblasts [24].

Connective tissue contracture is a low-energy, shortening process which involves matrix-dispersed cells and is dominated by extracellular events such as matrix remodeling [25]. Once achieved, contracture shortening does not require the continuing action of MFB as the shortened ECM restrains the surrounding tissues. This represents a “slip and ratchet” theory for contracture [26]. It might be speculated that the initial increased stiffness of the healing ECM at the socket level, with resident cells locking tension into the collagen structure in a interstitial, incremental manner might pose a greater risk of resorption for the thin vestibular bone plate. That would also partially explain why the greater part of tissue remodeling occurs at the early stages of healing.

The use of a free gingival graft could act as a tent over a tensioned structure: the fragile periosteum could be supported by the tent gently pulling at the top of the alveolus, preventing the collapse of the walls with inevitable wound contraction. Karaca and colleagues suggested that the use of a free gingival graft to cover the orifice of the socket could preserve bone height following extraction [27]_._

It is well known that tissue repair and remodeling depend on the bulk thickness of in vivo tissues and on the topological and mechanical features of the wound. Cells crawling and contracting towards a specific direction depending on differential gradients in ECM stiffness is a process referred to as durotaxis [24]. Durotaxis of FB and MFB in the extraction wound might be tuned with surgical artifices, such as the socket sealing technique, as hypothesized in the present study.

Both the size and shape of the wound have important effects on the resulting healing: the larger the wound, the more tissue displacement is needed to close it, and the more prominent would be the resulting scar. No matter the temporal similarities between the rabbit model and human socket healing, the rabbit alveolus is so small that this might account for the little difference observed between the two groups in the present study. The small rabbit incisor alveolus might have been too weak a model to find differences related to surgical management of the wound, thus giving rise to low inter-group test sensibility [28]. 

In conclusion, the present study implemented a type of molecular analysis which is new to oral sciences: a wide custom gene panel was defined and processed using high-throughput Nanostring technology, aiming at characterizing the expression profile related to myofibroblast activation after tooth extraction. The results suggest that myofibroblast activity is strongly exhibited during the early stages of healing. This time-related behavior of myofibroblasts might help in defining better socket preservation techniques and post-extractive implant positioning strategies. Further studies are needed to confirm this hypothesis.

## Figures and Tables

**Table 1:** The custom gene panel including 148 target genes and 15 housekeeping genes.

**Myofibroblast-Activation-Related Genes**
*ACTA1, COL1A1, COL1A2, COL2A1, CDH2, CDH11, S100A4, TAGLN, VIM, FN1, TNS4, TNS2, tensin-3-like, TNS1, MYH11, VCL, PXN*
**Myofibroblast-Inducing Genes**
*SMAD3, SMAD2, MECP2, HMGA2, SRF, TEAD4, SP1, SP3, CEBPB, CSl, c-myb, MRTF-A (MKL1), MRTF-B (MKL2), FlII, TRPV1, TNC, IL6, TEF*
**Autophagy-Related Genes**
*ATG8 (GABARAPL2), P62 (SQSTM1), AKT2, AKT3, mTOR*
**Myofibroblast-Suppressing Genes**
*SMAD7, NFKB1, KLF4, PPARG, NKX2-5, IL1A*
**Growth Factors and Cytokines**
*TGFB1, NREP, WNT3, JAG1, PTK2 (FAK), NOX4, IFNG, CXCL10, PDGFA, PDGFC, HAS2, PDGFRA*
**Genes Coding for ECM Components**
*ROS1, LOX, LOXL2, PLOD1, PLD2, SPP1 (OPN), POSTN, CTGF, EDA, Egr1*
**Genes Coding for Surface Proteins**
*CD248 molecule, ITGA11, ITGB1, ITGA3, ITGAV, ITGB5, ITGA5*
**Constitutive Expressed Genes in Normal Oral Mucosa**
Adhesion molecules: *CTNNA1, CTNNA2, CTNNA3, DCN, CDH5, NECTIN1, ITGA6* Chemokine/cytokine/growth factors: *S100A9, S100A8, TMSB10, TMSB4X, CCL5, ACE, KITLG, INHBA, PTMS, GPI* Receptor: *IL1R2, HBEGF, interleukin-2 receptor subunit alpha* Cell cycle/apoptosis: *RPS19, CDK6, PTMA, RPS3A, DYNC1H1, CAPNS1, APAF1, CAPN2, DAD1* Metabolism: *AQP3* Protease/protein turnover: *APP, PRNP, SERPINB5, IGFBP2, SERPINB1, CTSD* Signal transduction: *DBI, RHOA, SFN, JUP, S100P, protein S100-A7-like, CTTN, NTF3, PTPRF, MAP2K6, NME1, ARHGDIB, YWHAB, RAB2A* Transcription: *MLH1, GNAS, RPL6, CSDC2, ZFP36L1, KLF5, YBX1, ATF4, NUCB1, CNBP, RAD23B, SUB1* Homeostasis & detoxification: *HSPB1, GSTP1, HSP90AA1*
**Deregulated Genes at 48 h Post-Surgery**
Chemokine/cytokine/growth factors: *CCL2* Protease/protein turnover: *MMP3, MMP11, TIMP1* Signal Transduction: *CXCR1, MAL* Metabolism: *APOE* Transcription: *ID2*
**Housekeeping Genes**
RPL13A, UCHL-1, GAPDH, TUBA1B, ACTB, RPS9, ACTA2, HMBS, HPRT1, LDHA, TBP, NONO, EEF1E1, PPIH, PPIA

**Table 2:** Differentially expressed genes involved in the activation of myofibroblasts.

Myofibroblast-Activation-Related Genes												
Genes	*p*-Values	T1 vs. T2	T1 vs. T3	T1 vs. T4	T1 vs. T5	T1 vs. T6	T2 vs. T4	T2 vs. T5	T2 vs. T6	T3 vs. T4	T3 vs. T5	T3 vs. T6
***CDH11***	**0.0183**	0.042	0.006	0.089	0.221	0.811	0.743	0.420	0.023	0.172	0.079	**0.003** **Up**
***COL1A1***	**0.0093**	0.052	**0.002** **Up**	0.029	0.121	1.000	0.811	0.698	0.052	0.196	0.071	**0.002** **Up**
***TAGLN***	**0.0096**	0.438	0.330	0.199	0.310	0.018	0.039	0.073	**0.002** **Up**	0.043	0.071	0.004
***TNS1***	**0.0240**	0.095	0.021	**0.002** **Up**	0.004	0.023	0.161	0.221	0.550	0.845	0.961	0.643
***TNS2***	**0.0081**	0.189	**0.001** **Up**	0.006	0.039	0.009	0.152	0.455	0.189	0.242	0.084	0.205
***TNS4***	**0.0006**	0.511	0.922	0.007	0.025	**0.0002** **Up**	0.042	0.114	**0.003** **Up**	0.022	0.054	**0.002** **Up**

**Table 3:** Differentially expressed genes involved in the induction of myofibroblasts.

Myofibroblast-Inducing Genes												
Genes	*p*-Values	T1 vs. T2	T1 vs. T3	T1 vs. T4	T1 vs. T5	T1 vs. T6	T2 vs. T4	T2 vs. T5	T2 vs. T6	T3 vs. T4	T3 vs. T5	T3 vs. T6
*CSl*	**0.0032**	0.101	**0.001** **Up**	**0.002** **Up**	0.025	**0.001** **Up**	0.128	0.550	0.114	0.450	0.131	0.480
*HMGA2*	**0.0009**	0.858	0.380	0.013	**0.003** **Up**	**0.001** **Up**	0.021	0.005	0.010	0.252	0.124	0.060
*IL6*	**0.0003**	0.220	0.340	**0.0004** **Up**	**0.0004** **Up**	**0.0003** **Up**	0.023	0.024	0.020	0.057	0.058	0.052
*MECP2*	**0.0011**	0.144	0.088	**0.0001** **Up**	**0.002** **Up**	**0.001** **Up**	0.020	0.101	0.052	0.165	0.407	0.283
*MRTF-B (MKL2)*	**0.0096**	0.107	0.034	**0.001** **Up**	0.005	0.005	0.064	0.221	0.244	0.480	0.845	0.884
*SMAD2*	**0.0008**	0.339	0.283	**0.0002** **Up**	**0.003** **Up**	**0.002** **Up**	0.006	0.039	0.027	0.051	0.165	0.131
*SMAD3*	**0.0253**	0.069	**0.003** **Up**	**0.003** **Up**	0.022	0.037	0.244	0.633	0.788	0.609	0.283	0.214
*SP1*	**0.0008**	0.420	0.075	**0.0001** **Up**	0.004	0.020	**0.003** **Up**	0.039	0.020	0.196	0.575	0.435
*SP3*	**0.0051**	0.083	0.068	**0.0001** **Up**	0.005	0.004	0.042	0.282	0.244	0.214	0.643	0.592
*SRF*	**0.0226**	0.095	**0.001** **Up**	0.064	0.765	0.199	0.858	0.170	0.698	0.093	**0.003** **Up**	0.032
*TEF*	**0.0007**	0.633	0.048	**0.002** **Up**	0.011	**0.0003** **Up**	0.007	0.039	0.020	0.542	0.922	0.330
*TRPV1*	**0.0293**	0.009	**0.001** **Up**	0.079	0.105	0.119	0.400	0.328	0.298	0.076	0.059	0.053

**Table 4:** Differentially expressed genes coding for surface proteins.

Genes Coding for Surface Proteins												
Genes	*p*-Values	T1 vs. T2	T1 vs. T3	T1 vs. T4	T1 vs. T5	T1 vs. T6	T2 vs. T4	T2 vs. T5	T2 vs. T6	T3 vs. T4	T3 vs. T5	T3 vs. T6
*ITGA11*	**0.0311**	0.022	**0.002** **Down**	0.039	0.101	0.339	0.811	0.511	0.179	0.150	0.075	0.019
*ITGA3*	**0.0121**	0.339	0.075	0.045	0.698	0.325	0.296	0.179	0.976	**0.001** **Down**	0.144	0.010
*ITGAV*	**0.0374**	0.027	0.017	**0.002** **Down**	0.029	0.009	0.403	0.976	0.698	0.922	0.542	0.789
*ITGB5*	**0.0141**	0.049	0.014	**0.0003** **Down**	0.012	0.034	0.114	0.591	0.881	0.661	0.679	0.465
*KLF4*	**0.0095**	0.037	0.018	0.009	0.034	**0.0001** **Down**	0.612	0.976	0.101	0.807	0.526	0.495

**Table 5:** Differentially expressed genes coding for constitutive proteins in normal oral mucosa.

Constitutive Expressed Genes in Normal Oral Mucosa												
Genes	*p*-Values	T1 vs. T2	T1 vs. T3	T1 vs. T4	T1 vs. T5	T1 vs. T6	T2 vs. T4	T2 vs. T5	T2 vs. T6	T3 vs. T4	T3 vs. T5	T3 vs. T6
*S100P*	**0.0017**	**0.001** **Down**	**0.0004** **Down**	0.006	0.004	0.136	0.550	0.654	0.064	0.196	0.242	0.021
*ACE*	**0.0072**	0.325	0.407	0.121	0.121	0.045	0.011	0.011	**0.003** **Down**	0.036	0.036	0.014
*APP*	**0.0144**	0.012	**0.003** **Down**	0.009	0.069	0.438	0.929	0.492	0.083	0.394	0.137	0.019
*CCL2*	**0.0021**	0.095	0.845	0.014	**0.003** **Down**	**0.0003** **Up**	0.438	0.179	0.056	0.071	0.023	0.006
*CDK6*	**0.0105**	0.232	0.884	**0.002** **Down**	0.034	0.009	0.049	0.355	0.152	0.015	0.113	0.046
*CNBP*	**0.0004**	0.531	0.144	**0.001** **Down**	0.004	**0.0002** **Down**	0.005	0.023	**0.003** **Down**	0.180	0.367	0.131
*CTNNA1*	**0.0010**	0.199	0.733	**0.001** **Down**	0.016	**0.0004** **Down**	0.034	0.257	0.025	0.015	0.102	0.011
*CTNNA2*	**0.0276**	0.012	**0.002** **Down**	0.091	0.016	0.132	0.403	0.903	0.308	0.080	0.243	0.057
*CTTN*	**0.0040**	0.039	0.330	**0.001** **Down**	0.009	**0.0003** **Down**	0.221	0.571	0.128	0.088	0.242	0.051
*DBI*	**0.0040**	0.232	0.015	0.004	0.007	**0.0004** **Down**	0.089	0.128	0.020	0.942	0.826	0.661
*DCN*	**0.0126**	0.078	0.012	**0.001** **Down**	0.009	0.101	0.089	0.403	0.905	0.751	0.697	0.242
*DYNC1H1*	**0.0142**	0.037	0.262	**0.001** **Down**	0.014	0.005	0.179	0.720	0.455	0.093	0.380	0.232
*HBEGF*	**0.0366**	0.811	0.232	0.269	0.005	0.170	0.179	**0.003** **Up**	0.107	0.770	0.283	0.942
*HSPB1*	**0.0004**	0.189	0.981	0.005	**0.002** **Down**	**0.0001** **Down**	0.128	0.083	0.013	0.022	0.014	0.020
*IGFBP2*	**0.0001**	0.531	0.807	0.003	**0.0001** **Down**	**0.003** **Down**	0.022	0.020	0.017	0.032	0.004	0.027
*JUP*	**0.00101**	0.199	0.733	**0.002** **Down**	0.017	**0.001** **Down**	0.078	0.269	0.039	0.005	0.022	**0.002** **Down**
*KLF5*	**0.0004**	0.591	0.643	0.004	0.029	**0.001** **Down**	0.018	0.101	0.005	0.005	0.025	**0.001** **Down**
*MAP2K6*	**0.0012**	0.199	**0.003** **Down**	**0.0001** **Down**	0.009	0.004	0.011	0.179	0.107	0.922	0.380	0.510
*MLH1*	**0.0034**	0.257	0.157	**0.0001** **Down**	0.014	**0.002** **Down**	0.012	0.189	0.052	0.119	0.559	0.273
*NECTIN1*	**0.0002**	0.387	0.609	**0.001** **Down**	0.034	**0.001** **Down**	0.018	0.210	0.009	**0.002** **Down**	0.025	**0.001** **Down**
*NME1*	**0.0328**	0.511	0.542	0.025	0.073	**0.003** **Down**	0.114	0.257	0.020	0.223	0.394	0.068
*NUCB1*	**0.0198**	0.018	**0.001** **Down**	0.029	0.095	0.325	0.858	0.492	0.170	0.137	0.057	0.014
*PRNP*	**0.0077**	0.511	0.071	0.013	**0.002** **Down**	0.005	0.069	0.014	0.029	0.826	0.465	0.609
*PTMA*	**0.0293**	0.009	**0.001** **Down**	0.079	0.105	0.119	0.400	0.328	0.298	0.076	0.059	0.053
*PTPRF*	**0.0005**	0.161	0.592	**0.0003** **Down**	0.017	**0.0001** **Down**	0.032	0.325	0.016	0.018	0.157	0.010
*RAB2A*	**0.0144**	0.029	0.022	**0.001** **Down**	0.005	0.008	0.210	0.531	0.633	0.609	1.000	0.903
*RAD23B*	**0.0121**	0.069	0.318	**0.001** **Down**	0.009	**0.003** **Down**	0.152	0.420	0.232	0.097	0.252	0.144
*RPL6*	**0.0012**	0.743	0.023	**0.002** **Down**	0.005	**0.002** **Down**	0.005	0.014	0.007	0.770	1.000	0.826
*RPS19*	**0.0005**	0.339	**0.002** **Down**	**0.002** **Down**	**0.001** **Down**	**0.001** **Down**	0.029	0.023	0.013	0.592	0.643	0.770
*RPS3A*	**0.0003**	0.952	0.041	**0.001** **Down**	**0.002** **Down**	0.009	0.010	0.020	0.010	0.450	0.609	0.922
*SERPINB1*	**0.0069**	0.387	0.009	0.003	0.025	**0.002** **Down**	0.039	0.170	0.029	0.826	0.435	0.903
*SFN*	**0.0077**	0.339	0.592	0.034	0.032	**0.003** **Down**	0.244	0.232	0.042	0.023	0.022	0.030
*SUB1*	**0.0041**	0.049	**0.0002** **Down**	**0.003** **Down**	0.004	0.056	0.310	0.355	0.952	0.232	0.205	0.038
*TMSB4X*	**0.0293**	0.009	**0.001** **Down**	0.079	0.105	0.119	0.400	0.328	0.298	0.076	0.059	0.053
*YWHAB*	**0.0125**	0.189	0.097	**0.002** **Down**	0.008	**0.002** **Down**	0.078	0.179	0.073	0.394	0.609	0.380
*ZFP36L1*	**0.0056**	0.455	**0.001** **Down**	0.004	0.016	0.095	0.034	0.095	0.355	0.367	0.205	0.061

**Table 6:** Differentially expressed genes coding for ECM components.

Genes Coding for ECM Components												
Genes	*p*-Values	T1 vs. T2	T1 vs. T3	T1 vs. T4	T1 vs. T5	T1 vs. T6	T2 vs. T4	T2 vs. T5	T2 vs. T6	T3 vs. T4	T3 vs. T5	T3 vs. T6
*ROS1*	**0.0198**	0.016	**0.001** **Down**	0.188	0.023	0.142	0.271	0.890	0.343	0.025	0.147	0.035

**Table 7:** Differentially expressed genes coding for growth factors and cytokines.

Growth Factors and Cytokines												
Genes	*p*-Values	T1 vs. T2	T1 vs. T3	T1 vs. T4	T1 vs. T5	T1 vs. T6	T2 vs. T4	T2 vs. T5	T2 vs. T6	T3 vs. T4	T3 vs. T5	T3 vs. T6
*JAG1*	**0.0004**	0.244	0.751	**0.0001** **Down**	0.009	0.009	0.008	0.152	0.144	**0.001** **Down**	0.015	0.014
*NOX4*	**0.0407**	0.083	**0.002** **Down**	0.045	0.161	0.492	0.788	0.743	0.296	0.144	0.051	0.011
*NREP*	**0.0128**	0.128	0.008	0.078	0.144	0.531	0.811	0.952	0.032	0.223	0.144	**0.002** **Up**
*PDGFA*	**0.0257**	0.032	**0.003** **Down**	0.098	0.446	0.633	0.622	0.165	0.095	0.095	0.016	0.008
*PTK2 (FAK)*	**0.0169**	0.018	0.043	**0.001** **Down**	0.013	0.005	0.269	0.905	0.676	0.421	1.000	0.807

**Table 8:** Differentially expressed autophagy-related genes.

Autophagy-Related Genes												
Genes	*p*-Values	T1 vs. T2	T1 vs. T3	T1 vs. T4	T1 vs. T5	T1 vs. T6	T2 vs. T4	T2 vs. T5	T2 vs. T6	T3 vs. T4	T3 vs. T5	T3 vs. T6
*ATG8*	**0.0276**	0.012	**0.002** **Down**	0.091	0.016	0.132	0.403	0.903	0.308	0.080	0.243	0.057
*mTOR*	**0.0026**	0.032	0.751	0.018	0.152	**0.0001** **Down**	0.834	0.474	0.083	0.108	0.394	0.004
*P62*	**0.0008**	0.095	0.330	**0.001** **Down**	**0.003** **Down**	**< 0.0001** **Down**	0.121	0.199	0.018	0.097	0.150	0.021

**Table 9:** Differentially expressed genes deregulated at 48 h post-surgery.

Deregulated Genes at 48 h Post-Surgery												
Genes	*p*-Values	T1 vs. T2	T1 vs. T3	T1 vs. T4	T1 vs. T5	T1 vs. T6	T2 vs. T4	T2 vs. T5	T2 vs. T6	T3 vs. T4	T3 vs. T5	T3 vs. T6
*APOE*	**0.0094**	0.007	**0.003** **Down**	0.005	0.121	0.339	0.905	0.257	0.083	0.465	0.079	0.025
*CXCR1*	**0.0033**	0.221	0.010	0.010	**0.001** **Up**	**0.001** **Up**	0.174	0.049	0.028	0.652	0.961	0.817
*ID2*	**0.0013**	0.455	0.084	**0.000** **Down**	0.018	**0.002** **Down**	**0.003** **Down**	0.107	0.020	0.180	0.845	0.435
*MAL*	**0.0021**	0.310	0.626	0.083	0.025	**0.000** **Down**	0.474	0.221	0.012	0.057	0.021	**0.001** **Down**
*MMP3*	**0.0005**	0.511	0.092	**0.002** **Up**	**0.0004** **Up**	**0.001** **Up**	0.014	0.004	0.007	0.386	0.241	0.288
*TIMP1*	**0.0010**	0.511	0.380	0.006	**0.003** **Up**	**0.0001** **Up**	0.037	0.022	**0.003** **Up**	0.172	0.125	0.038
